# It’s All in the Interaction: Quantitating Gene Networks

**Published:** 2004-08

**Authors:** Carol Potera

Toxicologists who use microarrays hope to uncover relationships that link gene expression data to signal transduction pathways, gene networks that are often used to describe the sequence of biochemical events controlling cellular function. The large quantities of data generated by microarray studies generally are examined qualitatively—for example, by comparing whether one gene is turned on relative to another. These qualitative relationships, however, fail to describe how genes in a network influence each other. Still in their infancy are tools that quantitate the complex relationships within gene networks more comprehensively than simple correlations between pairs of genes. Now, for the first time, researchers describe a new quantitative statistical technique that assesses the interactions of genes in a network **[*EHP* 112:1217–1224].**

The team, led by Hiroyoshi Toyoshiba of the NIEHS Laboratory of Computational Biology and Risk Assessment, created a statistical software program that verifies concurrently that the expression of one gene is linked to the expression of several others. The first proof-of-concept demonstration evaluated genes that are directly responsive to tetrachlorodibenzo-*p*-dioxin (TCDD; a ubiquitous environmental pollutant and known human carcinogen) and their effect on the retinoic acid signal transduction pathway.

Signal transduction pathways respond to different environmental conditions; they are like molecular circuits that detect and integrate diverse external signals to alter gene transcription. This results in changes in enzyme activities as well as the production of abnormal levels of proteins, which further results in changes in biochemical processes. Alterations in signal transduction pathways can lead to cancer and other disorders.

Toyoshiba and colleagues had earlier identified genes that are altered in lung airway epithelial cells after exposure to TCDD. Starting with microarrays composed of 2,000 genes that are known to be expressed in response to environmental toxicants, the researchers had identified 11 genes that responded significantly to TCDD in two different lung cell lines. These genes appeared to be involved in the effects of TCDD on the retinoic acid signal transduction pathway.

The researchers constructed a hypothetical model of the retinoic acid signal transduction pathway that describes how the 11 genes interrelate. Based on published reports on retinoic acid metabolism, the model postulated that dietary vitamin A (retinol) is converted first to retinal and then to retinoic acid by alcohol dehydrogenases and, possibly, by cytochrome P450 enzymes. Once synthesized, retinoic acid enters the cell nucleus. There, it binds retinoic acid receptor beta, which, in turn, alters the expression of genes that may play a role in tumor formation. The hypothetical model included genes that produce three alcohol dehydrogenases, a cytochrome P450 enzyme, retinoic acid binding proteins and receptors, and four nuclear proteins.

Following exposure to three concentrations of TCDD, the expression levels of the 11 genes were calculated relative to unexposed controls. Statistical methods were applied to these data to test the hypothetical linkages between TCDD-responsive genes and the retinoic acid signal transduction pathway. These tests confirmed strong linkages between the genes included in the hypothetical model.

Epidemiological studies show a strong association between TCDD and lung cancer; the model offers a potential explanation for how TCDD damages the lungs. TCDD appears to activate genes associated with the synthesis of retinoic acid, which—through the retinoic acid signal transduction pathway—turns on nuclear genes that promote cell proliferation and carcinogenesis. Scientists can focus future experiments on particular genes directly related to TCDD-induced tumor progression.

The new statistical tool makes it possible to understand biological pathways in cells, tissues, organs, and whole organisms. It can be expanded to include other relevant data, such as protein levels in cells. These data can be combined with pharmacological models to present a true systems biology approach to quantifying risks from exposure to xenobiotics such as TCDD, suggest the authors. Other researchers can obtain the statistical software by contacting laboratory director Christopher Portier at portier@niehs.nih.gov.

## Figures and Tables

**Figure:**
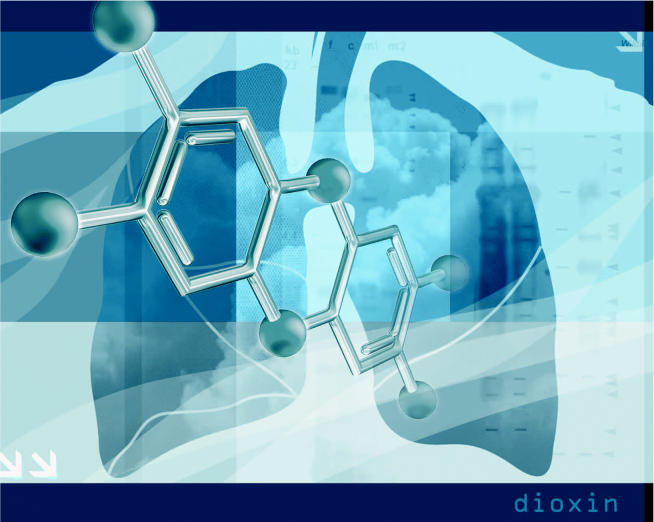
**Notating networks.** A new statistical package goes beyond qualifying interactions between a single pair of genes to describe how multiple genes within a network influence expression.

